# Health care provider perspectives on pregnancy and parenting in HIV-positive individuals in South Africa

**DOI:** 10.1186/1472-6963-14-384

**Published:** 2014-09-12

**Authors:** Jennifer Moodley, Diane Cooper, Joanne E Mantell, Erin Stern

**Affiliations:** Women’s Health Research Unit, School of Public Health & Family Medicine, University of Cape Town, Anzio Road, Observatory, Cape Town, 7925 South Africa; HIV Center for Clinical and Behavioral Studies, Division of Gender, Health and Sexuality, New York State Psychiatric Institute and Columbia University, New York, NY USA

**Keywords:** HIV-positive, Providers, Fertility intentions, Safer conception, Adoption, Parenting, South Africa

## Abstract

**Background:**

Within the health system, limited attention is given to supporting the fertility and parenting desires on HIV-positive people. In this study, we explore health care providers’ knowledge and perspectives on safer conception and alternate parenting strategies for HIV-positive people.

**Methods:**

Between November 2007 and January 2008, in-depth interviews were conducted with 28 health care workers involved in providing HIV and/or antiretroviral services at public sector clinics in Cape Town, South Africa. Views on sexual and reproductive health services, pregnancy, childbearing and parenting in HIV-positive men and women were explored using a semi-structured interview guide. Data were analyzed using a thematic approach.

**Results:**

Providers recognized the sexual and reproductive rights of HIV-positive individuals, but struggled with the tension between supporting these rights and concerns about spreading infection. Limited knowledge of safer conception methods constrained their ability to counsel and support clients in realizing fertility desires. Providers believed that parenting alternatives that do not maintain biological and cultural linkage are unlikely to be acceptable options.

**Conclusions:**

Health care provider training and support is critical to providing comprehensive sexual and reproductive health care and meeting the fertility desires of HIV-positive people.

## Background

With the rapid rollout of antiretroviral services in sub-Saharan Africa, the face of the HIV epidemic is rapidly changing. By the end of 2012, an estimated 9.7 million people in low- and middle-income countries had access to antiretroviral therapy (ART) [1]. In recent years, HIV-related mortality and morbidity have declined, the quality of life has greatly improved, HIV is now viewed as a chronic condition, and the vast majority of people living with HIV are of childbearing age. This combination of factors has contributed to a greater awareness of the sexual and reproductive health (SRH) needs of HIV-positive individuals; however, attention to their safer conception needs, i.e., reducing infection risk while trying to conceive and non-biological forms of parenting, have lagged behind their contraceptive needs [2].

Fertility in Africa is highly prized, with strong societal pressures to have children, the need to have boys as heirs, and preference for large families [3–5]. Research indicates that childlessness is stigmatized [6–8]. Numerous studies in Africa and elsewhere have shown that many HIV-positive women and men desire children [6, 9–11]. This desire is influenced by a number of factors, including a partner’s HIV status and fertility desire [12], feeling clinically well [10], having adequate socio-economic support [10], cultural beliefs [6] and being on ART [9, 13, 14]. Studies have shown that ART use is associated with higher pregnancy rates among HIV-positive women [9]. A substantial proportion of HIV-positive people become parents, underscoring the need to address issues of safer conception. Enabling HIV-infected couples to embark on the pathway to safer childbearing and non-biological parenting options has important health service implications. Although technology-intensive safer conception options such as intrauterine insemination and intracytoplasmic sperm injection are beyond the resource capacity of most developing countries, strategies, such as assisted manual self-insemination, sperm washing, and timed intercourse, are less technology-intensive and could play a role in reducing HIV risk for infected individuals in resource-constrained settings such as South Africa.

Health care providers play a central role in influencing and regulating access to SRH services. Provider perceptions that HIV-positive individuals should not engage in sexual activity or have children can constrain health care-seeking behavior with potentially detrimental health consequences [15, 16]. In South Africa, little is known about providers’ knowledge and perspectives on safer conception and alternate parenting strategies, such as adoption, for HIV-positive individuals. This article reports on qualitative interviews that explored health care providers’ attitudes toward pregnancy, safer conception and adoption for HIV-positive individuals as part of formative research for the development and testing of a multi-level structural intervention integrating SRH issues into HIV care for HIV-positive women and men.

## Methods

### Study site

The study was conducted between November 2007 and January 2008 at six primary health care clinics in Cape Town, South Africa. Services provided at the clinics include general HIV care services and CD4 monitoring; ART; SRH services such as family planning, antenatal and post-natal health care, maternity services, termination of pregnancy counseling, sexually transmitted disease care and cervical cancer screening; treatment of communicable diseases such as tuberculosis; child care services; treatment of minor ailments; and treatment of chronic diseases (e.g., hypertension and diabetes). All health facilities are located in predominantly urban working class communities with high HIV prevalence and are broadly representative of primary care services available for HIV-positive individuals in this setting.

### Study design

In-depth interviews were conducted with 28 health care workers involved in providing HIV and/or ART services. Participants were recruited using purposive sampling. Interviews were conducted by two trained qualitative interviewers, in a private space within the participants’ place of work. A semi-structured interview guide was used to elicit and probe knowledge and views on SRH services and their integration into HIV care services as well as pregnancy, childbearing and parenting in HIV-positive men and women. Here, we limit analysis to participants’ attitudes about pregnancy, safer conception and alternative parenting strategies. Interviews were conducted in English, audio-recorded, and transcribed verbatim.

### Data analysis

Data were analyzed using thematic analysis, which is a useful strategy for revealing the prominent themes in the text and providing a rich and holistic account of the data [17]. Initial coding categories for analyzing data were drawn from the interview guides, with additional categories developed from emergent themes and patterns from the data. Over several meetings, three members of the research team developed and refined the codes using the key issues probed. Two transcripts were independently coded and codes were then discussed to ensure common interpretation. One team member then coded all transcripts based on the final codebook. Once all the text segments had been given basic codes, the codes were categorised into basic themes by placing similar codes together. Additional themes were created during this process, which required continually going back to the transcripts to select relevant latent meaning in the text. Coding discrepancies were resolved through discussion and consensus from all research team members. Codes were entered into NVivo 7, a standardized qualitative data software program used to facilitate data sorting and management.

### Ethics

The study protocol, interviews, and consent forms were reviewed and approved by the Institutional Review Board at the New York State Psychiatric Institute and Columbia University Department of Psychiatry, and the Health Sciences Faculty Human Research Ethics Committee at the University of Cape Town. Permission to conduct the study also was obtained from all relevant health service authorities. All participants completed written informed consent prior to being interviewed.

## Results

This section describes the study participants and knowledge and attitudes on the SRH needs of HIV-positive clients, pregnancy and termination of pregnancy, safer conception, adoptive parenting, and provider SRH training needs.

### Study participants

Twenty-eight health care providers were interviewed: 4 doctors, 20 nurses, 3 counselors and 1 general assistant. The majority (n = 24) were female and ranged in age from 25 to 55 years. Participants had been involved in providing HIV and/or SRH service for a median of 6.5 years (interquartile range = 4 to 10 years).

### Sexual and reproductive health needs

Many providers felt that the SRH needs of HIV-positive people were not unique – but similar to those of uninfected individuals. Providers responded that HIV-positive clients should live a normal life and have sexual intercourse, provided they use condoms. Others concurred but believed HIV-positive people faced additional challenges, especially consistent condom use among those in long-term partnerships.

Providers recognized the importance of talking about SRH with their clients; however, many lamented the lack of time to have proper in-depth discussions with clients. As one participant said:

*My dear, I don’t want to lie, even myself, I don’t sometimes, though I know I’m supposed to*. (Professional Nurse)

At the same time, many reported that clients rarely talk about sex openly or ask questions because it is not normative in the community to discuss this topic. Talking about sex with a client considerably older than the provider was seen as especially challenging. “You can’t go straight away and be direct” since this would be viewed as a sign of disrespect in Xhosa culture. One provider gave an example of an indirect means of learning if a client is sexually active — *when did you last time skip the fire*? This cultural barrier also operated in the case of a younger client and older provider. Other difficulties alluded to were male clients’ particular reluctance to have open discussions about SRH issues with health care providers and the lack of privacy due to space constraints in some health facilities.

### Views on pregnancy and termination of pregnancy

In general, providers felt that HIV-positive individuals had the right to have children. Although pregnancy decisions often were upheld as being a personal choice, not one that should be determined by the provider, policy or government, providers acknowledged the significant cultural pressures on women to conceive. The desire to bear a child, irrespective of HIV status, was not only seen as natural, but as essential to fulfill a marriage and to be a woman. However, concern was expressed about the health risks of a pregnancy to both the mother and child. Providers articulated their role as educating clients on these risks and advising optimal timing of a pregnancy in relation to the women’s immune status (i.e., high CD4 count and low HIV viral load). As one provider said:

*…the only thing that is needed, I think, to be stressed, is education and they need to be told if maybe they are on ARVs, that they need to look to their CD4 count - saying that your CD4 count is very low, and if you are going to have a baby, this and this and this and this will happen. So you need to wait until your CD4 count has risen up and your virus is lower than detectable, so that if maybe you are pregnant, even your child will have more chances of being negative at the end of the day. But if they are well, their CD4 count is high and they are healthy, I don’t think we need to, sort of to stop them not to do whatever. Ja, it is their right, to be pregnant or not.* (HIV Counselor)

Providers also mentioned the availability of social support systems and number of existing children as important considerations that should be part of HIV-positive individuals’ childbearing decisions.

*I think the age of the person would be an advantage, and the family, if both sets of parents are there, if grandparents are there and there’s support around the family and the couple also maintains a healthy lifestyle, I think – I think they could have a child or two, but you know, for a person that doesn’t have any family support and doesn’t have any financial support, then it becomes a problem.*

(Clinical Nurse Practitioner)

*I can say to them, they can have a baby if the person is not having one, at least, they can have one, but if someone is having 3 babies already, I can say to them – no man, it’s not okay to have another one. I can say to her – no man, how about you can go and close yourself* [tubal ligation] *for the sake that now you already have 3 babies. Because once you get pregnant and deliver the baby, your CD4 count’s going to drop and then you are going to be weak and all that stuff, so it’s not going to be okay for you, but it’s your choice.* (HIV Counselor)

For some providers, the risk to the child in terms of HIV acquisition and the possibility of an orphaned childhood provided justification to counsel against a pregnancy.

*I don’t think it’s a good thing, I really don’t think it’s a good thing, because it’s a high risk factor, you know, but I’ve seen people with HIV having babies, I’ve seen it, but I don’t think it’s a good thing. During labour, you know, the child can be infected during that process, okay, because of the bloods, the exposure of the bloods, ne, maybe apart from the treatment that she’s getting, the other treatment for prevention of HIV, but I think it’s a high risk on its own.* (Clinical Nurse Practitioner)

The importance of individuals being healthy enough to raise their children was seen as a reason for HIV-positive individuals to avoid childbearing.

*They can be sexually active, but what I don’t like… is for them to fall pregnant, that’s why we stress the family planning. I stress the condom thing because although they say they can fall pregnant if their CD4 counts are high or all those things, but to me, it’s worse if they’ve got children. I don’t want them to fall pregnant, because it doesn’t matter whether your CD4 count is high today, and then you fall pregnant and because of the stress of the pregnancy, you drop that CD4 count to zero and then who knows that you’re going to be able to pick it up again and then it’s going to be worse now, you’ll be leaving this baby now and then that baby, it doesn’t matter whether you’ve got a good mother, you’ve got a good aunt, but it’s not like raising your own child and that somebody won’t raise that child like you were going to raise that child.* (Professional Nurse)

Health care providers’ views on termination of pregnancy (TOP) mirrored their opinions about an HIV-positive individual’s right to be pregnant. TOP was viewed as a client’s choice, irrespective of HIV status, and as being a viable option for a client with an unintended pregnancy.

*Termination of pregnancy should actually be offered to anybody, even if she’s not HIV-positive and say, you’ve got the option. If they come and say I don’t know what to do with this pregnancy, then you give them all the different options and then the patient can weigh, can look at the risks and benefits and come up with what they want, so I’m not opposed to that, but it mustn’t be just like – just because you are HIV-positive you should be going for, you know like first on the list and then the other options are small – no, they must have the same weight.* (Doctor)

However, when asked whether TOP should be routinely offered to HIV-positive women, many providers did not support this, as they noted the availability of ART for pregnant women and that most babies of HIV-positive women were HIV-negative. Providers stressed the need to empower women and their partners to use condoms to preclude unintended pregnancy. Few providers mentioned that clients had asked about or requested TOP. Most thought that the client, not the provider, should take responsibility for raising this topic. In the context of many clients only finding out they were HIV-positive when they became pregnant, one nurse articulated the importance of discussing the option of either continuing or terminating a pregnancy.

*What I don’t want is to just tell them, that person about PMTCT as if you’re saying now continue with the pregnancy, you tell them, you don’t tell them that … if you want it can be terminated, that is medically, the pregnancy could be terminated, that is your choice. Because what if …she didn’t want that pregnancy now that she’s HIV-positive, because she’s dealing now with this shocking news, stress and everything, but I didn’t…give her the information that this could be terminated*….(Professional nurse)

Providers’ personal opinions for being against termination of pregnancy also surfaced but reportedly did not interfere with their ability to listen and counsel patients. Some believed that the option to terminate a pregnancy should not be abused and treated as a routine contraceptive method.

### Views on safer conception

Health care providers generally had minimal knowledge of alternative methods of reproduction for HIV-positive individuals, often were unaware of where patients could access safer conception services, and felt uninformed to provide answers. For example, a provider, though favorably disposed to HIV-positive people having a child, was concerned that an infected partner could infect or re-infect the other partner with different strains of the virus and was unaware of any way to prevent this. In discussions on self-insemination, some providers raised concerns about contamination of sperm prior to manual self-insemination and the HIV-positive individual or partner’s dexterity in using a syringe to insert the sperm. Only a few health care providers mentioned they had talked with clients about manual self-insemination with their partner’s sperm using a syringe. These providers reported that clients appeared to be accepting of the procedure.

*If your partner is HIV, and the client is positive, we normally say to them they mustn’t stop using condom, they can use condom, okay, they can use a tiny syringe, and draw the semen and the woman can insert the semen into the vagina in the lying position.* (Clinical Nurse Practitioner)

*… just from personal experience, the patients who are keen to have a child, they seem to be fine with it, so I don’t think there’s like stigma and they see it, once they understand that we are trying to help them and they understand that this will have the best outcome for themselves and the child, and then they’re more amenable to it.* (Doctor)

One provider was aware of sperm washing and mentioned that it was currently being done on an “experimental basis” at the tertiary level fertility clinic and would be available in the future as would the use of donor sperm where both partners are HIV-positive. Providers reported that few patients had enquired about safer conception methods and some mentioned that clients might view safer conception as “not natural”, a cultural taboo, and perhaps would take a while before it would become acceptable to them. One provider stressed that people in the community believe that “to have a child you need to have sexual intercourse”, and that in the case of insemination with donor sperm, “you don’t even know the father of the baby”. Providers also worried about the costs of safer conception to the health system. However, providers were interested in learning more and being able to share this information with their patients.

*I don’t know if they will accept it, our people, uh huh, our culture, because there’s this culture business of us, you must know from which clan is that child come from and the genes and the what, what*. (Professional Nurse)

### Views on adoptive parenting

Providers reported that it was uncommon for HIV-positive clients to raise adoption as a parenting option. Some health care providers were in favor of adoption as an alternative means of parenting for HIV-positive individuals, but recognized the responsibility associated with it:

*It’s very difficult because I think here you look at the best interests of the child. You know if we could guarantee that they were going to stay healthy and whatever, then it’s not a problem, but you can’t guarantee even with ARVs, because they fail but I think it must come with huge responsibility…people must really think hard and have, and it mustn’t be like for themselves only, but as we’re saying with the best interest of the child.* (Doctor)

However, most providers felt that clients want to have a biological child and would not be receptive to adoption. As one professional nurse said “You want to pass on your genes to someone, even if you die”. Other difficulties mentioned were cultural difficulties in raising a child from another clan and community members questioning the couples’ reasons for adoption, particularly in situations of non-disclosure of HIV status. Some providers mentioned the social ramifications of adoption.

*Well an adoption would be a good thing to do, but mmm, I would also say in our tradition, as Xhosa-speaking people, we are not used to adoption. I think your in-laws or whoever, people in the community would want to know, why can’t you bear children of your own, why, they would be interested in criticizing you, you know. But it’s a good thing to do, because you can give love to other children that are going, that are abandoned, you know.* (Counselor)

Or, as another provider noted,

*Some of people say there’s something like inimba* [isiXhosa term referring to deep feelings of love expected to be found in a woman by virtue of the fact that she’s gone through with a childbearing process]*, if you never carry that baby, then you won’t feel anything about, you won’t feel the love or whatsoever, because you never carried*… (Clinical Nurse Practitioner)

Providers noted that “informal” adoption, in which family members raise a blood relative’s child, is a normative community practice. In the providers’ opinion, familiarity with a child’s customary background and the potential for reciprocity made this a more desirable option than formal adoption. Providers also felt that there needed to be greater discussion about adoption as a parenting option with HIV-positive clients.

*Because in this community they don’t believe in adopting, but they do believe in taking my sister’s child and look after my sister’s child – but I don’t know what do they call that. They don’t want to say I’ve adopted my sister’s child, but they are saying – because my sister dies, I look after my sister’s child. Because they’ve got this thing, you bring somebody, like, like to us, you must – we’ve got clan names, so if you are Nguni, you don’t know what is happening to another clan, to Madiba clan, the way they are doing things the Madiba clan, it’s not the same, like the Nguni’s are doing things.* (Professional Nurse)

### Provider training needs

None of the providers reported receiving training on safer conception methods. Other training needs mentioned were contraception for HIV-positive clients and management of sexually transmitted infections. Providers emphasized the importance of ongoing training and recognized that lack of information hindered their ability to fully serve their clients.

*There is a need for training for those who, who don’t have much information and that, so that it can be able to empower them, our clients. So sometimes really, if you don’t have much information, it’s difficult for you to do*. (Professional Nurse)

Health care providers also noted the need for training to enhance their ability to speak openly about sex to clients. As one provider said:

*ja, like for instance, like, not to consider their feelings, you see like sometimes like, the way we were grown up, ne, you were in that situation that you mustn’t talk about sex, but now that we are health care providers, you mustn’t put your feelings first, you must know that, I have to give information, this is my job, I need to be open for this patient, you are helping this patient. It’s not about you, it’s about the patient*… (Professional Nurse)

## Discussion

The fertility desires of HIV-positive women and men are well documented [6, 9–11]. Notwithstanding this, assessment of fertility intentions is rarely addressed in public health care facilities. A study in Cape Town, South Africa, reported that more than one-third of women and two-thirds of men attending HIV care clinics were interested in having children, yet only 19% and 6%, respectively, had discussed their fertility desires with a health care provider [13]. Similar results have been reported in both developing and developed countries [10, 18]. International and national policies and guidelines focusing on the SRH needs of HIV-positive people are finally starting to address safer conception needs [19, 20]. The SA HIV Clinicians Society recently (2011) published safer conception guidelines for fertile HIV-positive individuals and couples [20]. Evidence from a range of studies in South Africa, however, have shown that health policies and guidelines intended to advance health and human rights objectives are often critically limited in their implementation by the skills, knowledge, beliefs and practices of health care providers [21–23]. For example, a Cervical Health Implementation Project that developed and evaluated health system interventions to improve public sector cervical cancer screening demonstrated the importance of improving provider skills and knowledge in implementing cervical screening guidelines [22]. Research has also shown how negative provider attitudes limited access to termination of pregnancy services in South Africa, despite the country’s liberal abortion laws [23]. In the context of cultural, health system and resource constraints, implementation of safer conception guidelines will be challenging. Our study provides invaluable information for preparing health care providers in the public sector to implement interventions that minimize HIV risk and support HIV-positive couples to realize their fertility intentions.

Health care providers and other health center workers are ‘gatekeepers’ to influencing whether services feel welcoming or judgmental [24]. Studies have shown that HIV-positive couples desiring conception often hesitate to reveal fertility plans to health care providers for fear of negative judgment [10, 18, 25]. Our study did not reveal discriminatory views by providers. Rather, health care providers reported recognizing and supporting the reproductive rights of all couples and individuals “to decide freely and responsibly the number, spacing and timing of their children and to have the information and means to do so,” as is articulated in the South African Constitution. This, together with the findings of recent survey in Durban, South Africa, where HIV-positive couples recognized health care providers as a resource for conception-related information and counseling [26], suggests a changing clinical environment. Nevertheless, even in qualitative interviews in which rapport is nurtured, reported attitudes and behavior may differ from reality and health care providers may have narrated their responses in support of expectations of the researchers. As the majority of providers were female, we were unable to explore gender differences in attitudes and how gender affected health care providers’ attitudes. However, some of the views expressed by providers related to norms of womanhood, such as the desire to bear a child, and may have been related to the largely female population of providers.

In our study, providers were cognizant of the sexual and reproductive rights of HIV-positive individuals but grappled with the inherent tension between supporting the right to have children and that of preventing HIV transmission to both the partner and unborn child. Biomedical considerations were paramount in providers’ approach to HIV infection and fertility desire; however, these considerations were often not based on accurate knowledge. Despite evidence that pregnancy does not speed progression of HIV disease [27–29], - some providers offered this as a reason for not supporting fertility desires of clients. Conception and pregnancy do confer risks of HIV transmission; however, these can be significantly reduced through specific health care interventions [20, 30]. The health care providers in our study were aware of optimal timing of conception in relation to a client’s immune status. A few providers were aware of manual vaginal insemination using a syringe, although some had concerns about the protective efficacy of the method. The majority of providers had limited clinical knowledge of safer conception methods and of their availability, constraining their ability to counsel and support clients in realizing their reproductive rights. This identified lack of knowledge offers a clear opportunity for intervention.

In our study, providers were aware of the cultural pressures associated with childlessness and appeared to find it easier to support couples or individuals with no children, compared to those with children. Research shows that some HIV- positive couples desire more than one child [11]. Reasons for this include wanting to have both male and female children, desire for large families and wanting to have children with new partners. Providers need to be aware of these influences on fertility decisions, if they are to provide the necessary support for clients.

Providers’ views on potential cultural difficulties with formal adoption in this setting echo those that have been articulated by HIV-positive clients in South Africa [31]. An extended family “informal adoption” arrangement is common practice in South Africa. This is not done through the legal system, but informally by family members without paper work and changes in surnames. This allows for cultural connection to be maintained. The most common scenario reported in our study was for the sister of the deceased mother to care for the orphaned child. Providers suggested that parenting alternatives and safer conception methods, such as insemination with donor sperm, that do not maintain biological and cultural linkage, are unlikely to be acceptable options in this context. In many cultures in sub-Saharan Africa, adoption is more of a family responsibility and duty rather than something that would be sought out, and could provide an acceptable alternative form of parenting for HIV-positive couples. The majority of providers interviewed were from a Xhosa cultural background. Cultural insights and understandings about difficulties of formal adoption in Xhosa culture and views of safer conception methods might relate to health care providers’ own cultural background.

Unlike quantitative research, qualitative research allows and encourages the process of self-reflexivity and acknowledges the researchers’ relationship and possible influence on the research process. In conducting this research, the researchers reflected on the divergence between their own backgrounds as researchers and those of the participants. Possible ways in which researcher perceptions impacted on roles and interpretations in the research process were discussed and taken into account in the data analysis and interpretation.

A limitation of our study, as with most qualitative research, is that of generalizability. Our study was conducted among public health care providers working at urban public sector health clinics in Cape Town and might not be applicable to non-urban or private sector settings. However qualitative studies seek to obtain a range of belief and attitude, rather than address generalizability. While it is possible that health care provider’s HIV status could have influenced their views on pregnancy, childbirth and adoption among HIV-positive people, we are unable to comment on this as provider HIV status data were not collected. This is an important issue to explore in future studies.

### Recommendations

To provide comprehensive reproductive health care to HIV-positive individuals, an investment in initial and ongoing health care provider training is vital. Training must include information on fertility intention assessment and on fertility options available for HIV-seroconcordant and serodiscordant couples. Providers are keen to support reproductive rights, but need to be trained to deal with cultural complexities and negotiate the dichotomy between supporting patient’s reproductive rights and preventing transmission of HIV to the patient’s child and/or partner. Values clarification training will help providers reflect on their own values and separate this from professional responsibilities. This could play an important role in transforming health care practices and in improving the quality of care and reproductive health outcomes of HIV- positive individuals. In addition to training, it is also important that assessment of reproduction intention become systematized into routine care, for example, by the use of standardized checklists. An enabling management, peer, practical and medical environment is also essential if any sustained changes are to occur. Limited research is available on provider attitudes and practices relating to childbearing among people living with HIV. In the context of increasing life expectancy of HIV-infected people, this research gap must be prioritized.

## Conclusion

In South Africa, access to antiretroviral treatment has rapidly expanded, with nearly 2 million South Africans currently on ART, transmission of vertical HIV transmission has been reduced to under 3 percent, the criteria for pregnant women receiving life-long ART has changed, and life-expectancy among HIV-infected individuals has increased [32, 33]. Given that the majority of people living with HIV/AIDS in South Africa are of childbearing age, managing fertility and safer conception must be seen as part of routine HIV care. Gathering insights from health care providers are an important step in in the delivery of comprehensive sexual and reproductive health counseling and service delivery
